# Lenalidomide monotherapy for necrobiotic xanthogranuloma

**DOI:** 10.1016/j.jdcr.2026.05.057

**Published:** 2026-06-03

**Authors:** Annabelle Huntsman, Erin McClure, Nicole Fett

**Affiliations:** a University of Utah School of Medicine, Salt Lake City, Utah; b Oregon Health and Science University, Portland, Oregon

**Keywords:** lenalidomide, monoclonal gammopathy, necrobiotic xanthogranuloma

## Introduction

Necrobiotic xanthogranuloma (NXG) represents an uncommon inflammatory non–Langerhans cell histiocytosis, often coexisting with monoclonal gammopathy.^1^ Given its rarity, there is no established standard of treatment, making NXG challenging to adequately manage. Here, we report a patient who demonstrated notable improvement in an ulcerated plaque and anemia following treatment with lenalidomide.

## Report of a case

A 70-year-old man with a history of pancytopenia, IgG-λ monoclonal gammopathy of undetermined significance, early-stage colon cancer status post hemicolectomy 10 years ago, and noncirrhotic portal hypertension due to nodular regenerative hyperplasia complicated by ascites presented to the dermatology clinic for evaluation of progressive skin lesions. Five years ago, the patient noticed “bumps” around his eyes. These were excised, but quickly recurred. Over time, these lesions spread to his trunk and extremities and began to ulcerate. The patient never experienced ocular lesions or symptoms.

Two prior punch biopsies of the patient’s lesions revealed large nodular aggregates of granulomatous inflammation throughout the superficial and reticular dermis. These aggregates were often found to be palisading around anuclear regions with degenerated collagen and cholesterol clefts.

**Fig 1:**
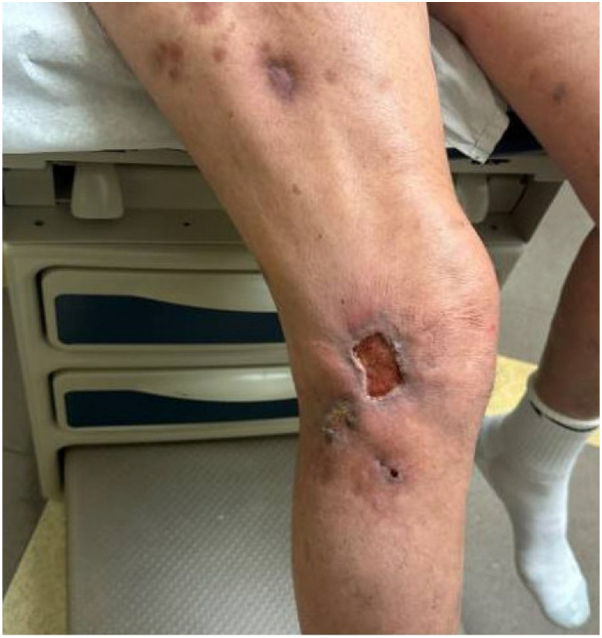
Diffuse yellow-brown depressed plaques and three ulcerations.

When the patient presented to the dermatology clinic, physical examination was notable for yellow-brown papules and plaques on the medial upper eyelids and infraorbital region, as well as scattered yellow-brown flat-topped papules on his trunk and extremities. On his right knee, a large ulceration was noted within a yellow-brown plaque, as shown in Fig 1. There was no evidence of eye inflammation or macroglossia.

The combination of the patient's monoclonal gammopathy of undetermined significance, pancytopenia, liver findings, skin findings, and pathology results supported NXG as a unifying diagnosis.

**Fig 2:**
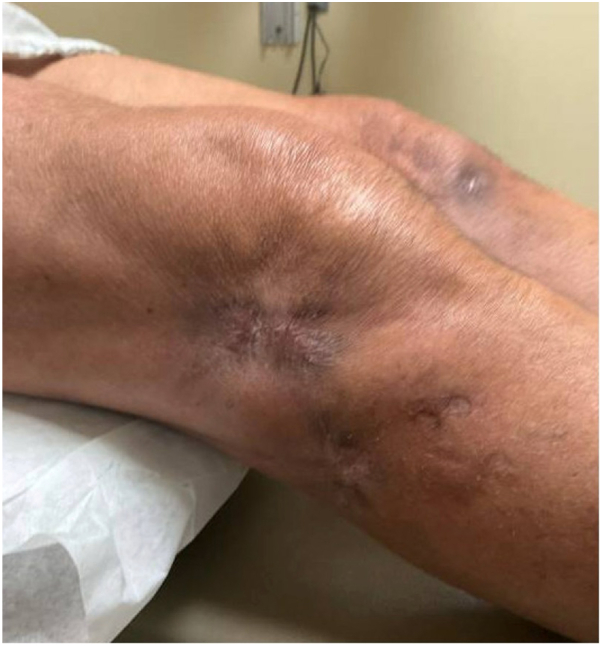
Healed ulcerations and less prominent yellow-brown depressed plaques.

Prior to presentation to the dermatology clinic, the patient was treated with prednisone 20 mg daily for ∼2 months, which was ineffective in improving his blood counts or cutaneous manifestations, and 3-monthly doses of intravenous immunoglobulin (IVIG, 1 g/kg), which he tolerated poorly with constitutional side effects and did not result in improvements of his skin or laboratory abnormalities. We started the patient on lenalidomide 5 mg daily. Notably, 4 months after beginning lenalidomide, the patient showed a marked clinical improvement, including resolution of his primary ulcer on his right knee and hemorrhagic-crusted erosions as demonstrated in Fig 2. Additionally, thinning of diffuse xanthomatous plaques, including his periocular lesions, was observed. Of note, anemia (prior hemoglobin level of 7.4 g/dL) also resolved (posttreatment hemoglobin level of 14 g/dL). Platelet and white blood cell counts remained stable at month 4.

**Fig 3:**
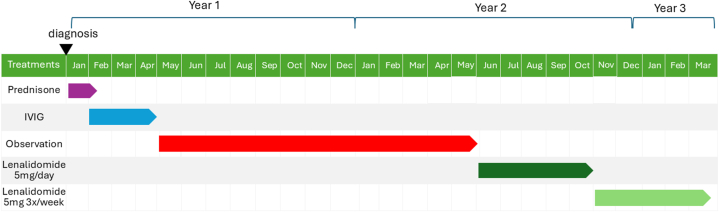
Treatment timeline.

The patient remained on 5 mg of lenalidomide for 5 months but then transitioned to 5 mg every other day due to leukopenia. Leukopenia is stable at this dose, but the patient has experienced new cutaneous ulcers. Lenalidomide may be increased later to 5 mg 4 to 5 times weekly depending on side effects and laboratory results. The patient’s lenalidomide treatment comprises his current maintenance therapy. His treatment timeline can be found in Fig 3. Total duration of treatment will be until the patient’s side effects become intolerable or lenalidomide is no longer satisfactorily efficacious. The patient has been followed for 9 months by his current dermatology team and continues to receive dermatologic follow-up every 3 months.

## Discussion

NXG is a rare non–Langerhans cell histiocytosis that initially presents clinically as yellow plaques periocularly. It is strongly associated with IgG monoclonal gammopathies and is often seen with plasma cell dyscrasias such as multiple myeloma, monoclonal gammopathy of undetermined significance, and other lymphoproliferative disorders.^2^ The pathogenesis is not well understood but thought to be related to paraproteins binding to lipoproteins and inducing xanthoma formation.^3^

Treatment options include IVIG, alkylating agents for patients with multiple myeloma, and systemic or intralesional corticosteroids, with variable efficacy.^2^ Pegylated interferon alfa-2a has recently been reported as an effective and well-tolerated treatment option for NXG.^4^ The largest systematic review to date, which analyzed 175 cases, found that corticosteroids and IVIG were the most frequently used and most effective therapies to treat NXG. Eighty-one percent (21 of 26) of patients had a complete or partial response after treatment with IVIG, and 67% (30 of 45) of patients experienced this with prednisone.^5^ Lenalidomide with or without steroids achieved response in 17 of 22 cases (77%). Notably, this study did not isolate lenalidomide monotherapy and instead merged these results with those of patients who also received corticosteroids.

Case reports have demonstrated that immunomodulatory agents such as lenalidomide have been used to successfully treat NXG.^6^, ^7^, ^8^ Consistent with these findings, our patient had demonstrated remarkable improvement of his ulcerative lesions just 4 months after beginning treatment with lenalidomide, with significant improvement in hemoglobin level. Although ulcers recurred when the dose was reduced, this may suggest a dose-dependent relationship on disease control.

Lenalidomide is an immunomodulatory agent that has anti-inflammatory effects, more specifically inhibiting the production of cytokine tumor necrosis factor-α, which may decrease granulomatous formation in patients with NXG.^9^ Lenalidomide has been shown to play a role in patients with myelodysplastic syndromes and demonstrated sustained independence from transfusion and increased hemoglobin levels, which suggest it may modulate ineffective hematopoiesis.^10^ Bone marrow biopsy revealed a normocellular marrow with normal trilineage hematopoiesis and no evidence of dysplasia. This is most likely attributable to resolution of underlying NXG-related inflammatory process rather than a primary hematologic disorder, although hemolysis was not specifically ruled out.

Cost is a consideration when selecting lenalidomide as a treatment option, especially for long-term management. In the United States, a 28-day cycle is ∼$18,700 to $25,000 but can vary depending on dosage, insurance, and pay assistance programs.^11^

Our findings support lenalidomide as a treatment option for NXG, particularly in patients with underlying monoclonal gammopathy or plasma cell dyscrasias.

## Conflict of interest

None disclosed.
